# Supplementary material for: Influences on limited antimicrobial use in small-scale freshwater aquaculture farms in central Thailand
Source: Front Vet Sci. 2025 Jul 11;12:1600051. doi: 10.3389/fvets.2025.1600051 (PMC12291687; doi:10.3389/fvets.2025.1600051)
Supplement: SUPPLEMENTARY APPENDIX S1 — Farmer survey. [file Data_Sheet_1.docx]

**Appendix S1. Famer survey**

(Questionnaire no.) …………………

(Date of data collection)…………………

**(Interview form for aquacultures)**

**(Part)1 (General information on respondent/farmer)**

1.1 (Age of respondent/farmer) …………

1.2 (Gender) ⬜ (Male) ⬜ (Female)

1.3 Education level

⬜ (Below primary school)

⬜ (Primary school)

⬜ (Secondary school)

⬜ (Bachelor)

⬜ (Above bachelor degree)

⬜ (Other, please specify) …...……….…………..

1.4 (Marital status) ⬜ (Single) ⬜ (Married) ⬜ (Divorced/separate)

(Number of children) …...……….…..... (please include ages of each child)………… …………………

(Number of people in your family live on the farm including yourself )…...……….….....

1.5 (Do any family members carry out work on the farm?) ⬜ (Yes) ⬜ (No)

1.6 (Does this work involve looking after the animals)

⬜ (Yes) ⬜ (No)

**(Part)2 (General information on farm)**

2.1 (Type of farm)

⬜ (Owned farm) ⬜ contract farm (with company)

⬜ (Other, please specify) …...……….…………..

(Number of full-time employees) ….…………………………

(Number of part-time employees) ………………………

(If the farm is owned, was the farm established by yourself or the farm bought or inherited?)

⬜ (Farm established by yourself) ⬜ (Farm bought)

⬜ (Farm inherited) ⬜ (Other, please specify) …...……

2.2 (Main animal species in the farm (more than one answer is allowed))

⬜ (Tilapia) (No. of animals) ………………

⬜ (Catfish) (No. of animals) ………………

⬜ (Snakeskin gourami) (No. of animals) ………………

⬜ (Other fish species, please specify) ……………… (No. of animals)………

⬜ (Pacific white shrimp) (No. of animals) ………………

⬜ (Giant freshwater prawn) (No. of animals) ………………

⬜ (Other shrimp/prawn species, please specify) …………………… (No. of animals) ………………

Note: “No. of animals” refers to ‘estimated number of animals of the year of interview’

2.3. (How often do you change the pond/tank water?)

⬜ (Monthly) ⬜ (every 2 months) ⬜ (every 6 months)

⬜ (annually) ⬜ (Other, please specify) …...……….…………..

2.4. (Where do you get water for use on your farm from?)

⬜ (River) ⬜ (Canal) ⬜ (tap/pipe) ⬜ (well/borehole)

⬜ (surface water (lake, pond))

⬜ (Other, please specify) …...……….…………..

2.5 Types and number of other animals in the farms (more than one answer is allowed)

⬜ (Chicken) (No. of animals) ……………

⬜ (Duck) (No. of animals) ………………

⬜ (Other species, please specify) …………… (No. of animals)………

2.6 (Detail on health)

| (topic) | (describe) |
| --- | --- |
| 2.6.1 (Numbers of veterinary visit the farm) | ⬜ (How many times does the vet regularly visit the farm in a year?…..)  ⬜ (How many times does the vet visit when something goes wrong in a year?…..)  ⬜ (How many times has the vet visited last year?……..) |
| 2.6.2 (Is there any farm management program?)  Hint: Who planned the herd health management program and what is it made up of? | ⬜ (Yes, if yes (voice record)  ⬜ (No) |
| 2.6.3 (Have you changed your management plan in the last three months and if so, why?) | ⬜ (Yes, if yes (voice record))  ⬜ (No) |
| 2.6.4 (Percentage of animal culling per production cycle) |  |
| 2.6.5 (Percentage of mortality per production cycle) |  |
|  |  |
| 2.6.6 (source of breeding stock) |  |
| 2.6.7 (Do you quarantine new entry?) |  |
| 2.6.8 (in the last 1 year, did your fish farm  face these problems?)   - (rotten wound) - (parasites) | ⬜ (found) ⬜  (not found)  ⬜ (found) ⬜  (not found) |
| 2.6.9 (in the last 1 year, did your prawn  farm face these problems?   - (Shrimp early mortality syndrome) - (protozoal disease) - (white prawn disease) - 69_White_feces_syndr | ⬜ (found) ⬜  (not found)  ⬜ (found) ⬜  (not found)  ⬜ (found) ⬜  (not found)  ⬜ (found) ⬜  (not found) |

2.7 (Total farm area (in Rai)) …………….

2.8 (Area of housing or animal production (in Rai))……………

- - (For main species (in Rai)) …………….
  - (For other species (in Rai))…………....

2.10 (How do you sell animals in your farms?)

⬜ (Sell direct to consumer)

⬜ (Sell to middleman)

⬜ (Sell to food company)

⬜ (Sell at fresh market)

⬜ (Other, please specify)………………….

**(Part) 3 (Data on antimicrobial consumption)**

3.1. (What type of antimicrobials or feed additives do you use?)

| (Generic name) | (Objectives)^1^  What other | (Drug preparation)^2^ | (Strength) | (Interval of administration) | (Weight of animals per Rai |
| --- | --- | --- | --- | --- | --- |
|  |  |  |  |  |  |
|  |  |  |  |  |  |
|  |  |  |  |  |  |
|  |  |  |  |  |  |
|  |  |  |  |  |  |
|  |  |  |  |  |  |
|  |  |  |  |  |  |
|  |  |  |  |  |  |
|  |  |  |  |  |  |
|  |  |  |  |  |  |

^1^ “ (“Objective” a: disease prevention, b: disease treatment, c: health promotor, d: growth promotor, e: other purpose)

^2^ (Drug preparation such as oral, parenteral, feed additive, etc.)

3.2 (Have you recently changed any doses or types of antimicrobials? Why and how?)

.…………………………………………………………………………………………………………….……………………………………………………………………………………………………………………………………………………….

3.3 5-10 (Do you think the use of antimicrobials is decreased or increased in the last 5-10 years? How?)

.………………………………………………………………………………………………………………………………… ………………………………………………………………………………………………………………………………….

3.4 (Expenses borne to the use of antimicrobials per year)

……………………………………………………………………………………………………………………………..…..

3.5. (What for you are the main reasons for using antimicrobials in your production?)

……………………………………………………………………………………………………………………………..…..

(Note): (Open question - but response might be categorized below)

(Main objectives of antimicrobial consumption)

⬜ (Disease prevention)

⬜ (Disease treatment)

⬜ (Health promotor)

⬜ (Growth promotor)

⬜ (Other purposes (specify))……………………………………………….

3.6 (In general, what is the health status of your animals? Can you estimate animal death rate in your farm?)

……………………………………………………………………………………………………………………………..…..

3.7 (Who determines type of antimicrobials used in your farm? When?)

……………………………………………………………………………………………………………………………..…..

3.8 (Did any animal disease outbreaks or mass dying recently occur in your farm?)

……………………………………………………………………………………………………………………………..…..

3.9 (If so (as stated in 3.8), when did the event occur? How did you manage? Did you use any antimicrobials in the treatment? Did you cull the animals?)

……………………………………………………………………………………………………………………………..…..

3.10 (In your production system, how much antimicrobials do you need to use? If you don’t use at the current amount, can you still produce the animals at your satisfactory level?)

……………………………………………………………………………………………………………………………..…..

3.11 (What would happen if you stopped using any antimicrobials?)

……………………………………………………………………………………………………………………………..…..

**(Source or antimicrobials and suggestions)**

3.12 (Where did you purchase antimicrobials or feed additives from?)

⬜ (Feed company)

⬜ (Veterinarian)

⬜ (Pharmaceutical manufacturer)

⬜ (Other, please specify)……………………………………………

3.13 (How do you use antimicrobials in animal feed?)

⬜ (Mix antimicrobials with the feed at the farm)

⬜ (Antimicrobials commercially mixed in the feed)

(If you purchase animal feed mixed with antimicrobials, who determines ingredients and amount of antimicrobials in the feed?)

……………………………………………………………………………………………………………………………..…..

(If you purchase animal feed mixed with antimicrobials, who determines amount of antimicrobials in the feed per raising area?)

………………………………………………………………………………………………………………………….

3.14 (What are the principal control systems and regulations for antimicrobials and feed additives for your farm? (more than one answer is allowed))

⬜ (The buying and purchasing of antimicrobials is controlled. )

(Who is it controlled by)……………………………………………………………………………

⬜ (The use of antimicrobials is controlled on the farm).

(Who is it controlled by)……………………………………………………………………………

⬜ (The buying control system of feed additives is controlled on the farm. )

(Who is it controlled by)…………………………………………………………………………

⬜ (Other, please specify)…………………………………………………………………...

3.15 (Do you feel you are being controlled with this control system)

………………………………………………………………………………………………………………………..

3.16 (How do you know which antimicrobials should be purchased?)

…………………………………………………………………………………………………………………………..

- (Did you learn or acquire any knowledge from your own trial-and-error and experience in the use of antimicrobials? How?)

……………………………………………………………………………………………………………………………..

- (From whom did you get advices on the sources of antimicrobials?)

…………………………………………………………………………………………………………………………..

3.17 (Have you heard about the impacts of antimicrobial use such as harmful effects on human, animal and environment from long term usage?)

…………………………………………………………………………………………………………………………..

**(Part) 4 (Antimicrobial storage)**

4.1 (Where do you keep antimicrobials on the farm?

…………………………………………………………………………………………………………………..

4.2 (Do you stock large amount of antimicrobials in your farm (especially feed additives with antimicrobials)? Where did you get these drugs?)

……………………………………………………………………………………………………………………………..

4.3. (Can we take a look at the drug storage in your farm? (Take photo if permitted))

……………………………………………………………

4.4 (Did you use all antimicrobials you have?)

……………………………………………………………………………………………………………………………..

4.5 (How did you manage waste and leftover antimicrobials?)

……………………………………………………………………………………………………………………………..

4.6 (Did you keep any records on the use of antimicrobials and dosages for animals in your farm?)

…………………………………………………………………………………………………………………………..

- (If so, do you keep the records systematically and continuously?)

……………………………………………………………… …………………………………………………..…..

- (Did you report the data to any authorities?)

……………………………………………………………………………………………………………………..…..

4.7 Can you identify individual treated animals? / How do you know which animals you have treated?

…………………………………………………………………………………………………………………………..

(Do you have any additional data or opinions?)

…………………………………………………………………………………………………………………………..
