# Supplementary material for: Influences on limited antimicrobial use in small-scale freshwater aquaculture farms in central Thailand
Source: Front Vet Sci. 2025 Jul 11;12:1600051. doi: 10.3389/fvets.2025.1600051 (PMC12291687; doi:10.3389/fvets.2025.1600051)
Supplement: SUPPLEMENTARY TABLE S1 — Farm characteristics. [file Table_1.docx]

**Supplementary Table S1. Farm characteristics**

| **ID** | **Age range** | **Gender** | **Education** | **Farm Type** | **Aqua species** |
| --- | --- | --- | --- | --- | --- |
| F1 | 80-89 | Male | Primary school | Farm established by the farmer | Tilapia, Pacific white shrimp, Giant freshwater prawn |
| F2 | 20-29 | Female | Bachelor | Farm established by the farmer | Tilapia, Pacific white shrimp, Giant freshwater prawn |
| F3 | 50-59 | Male | Secondary school | Farm established by the farmer | Tilapia, Probarbus Jullieni, Cyprinidae, Pacific white shrimp |
| F4 | 60-69 | Male | Primary school | Farm established by the farmer | Tilapia |
| F5 | 50-59 | Male | Secondary school | Farm established by the farmer | Tilapia |
| F6 | 60-69 | Male | Secondary school | Farm established by the farmer | Giant Gourami |
| F7 | 50-59 | Female | Primary school | Farm rented | Tilapia |
| F8 | 60-69 | Male | Primary school | Farm established by the farmer | Tilapia |
| F9 | 50-59 | Male | Primary school | Farm established by the farmer | Tilapia |
| S1 | 30-39 | Male | Secondary school | Farm established by the farmer | Tilapia, Pacific white shrimp, Giant freshwater prawn |
| S2 | 30-39 | Female | Bachelor | Farm established by the farmer | Pacific white shrimp, Giant freshwater prawn |
| S3 | 50-59 | Female | Primary school | Farm inherited | Pacific white shrimp, Giant freshwater prawn |
| S4 | 50-59 | Male | Secondary school | Farm established by the farmer | Pacific white shrimp, Giant freshwater prawn |
| S5 | 40-49 | Female | Secondary school | Farm inherited | Pacific white shrimp, Giant freshwater prawn |
| S6 | 60-69 | Male | Primary school | Farm rented | Pacific white shrimp, Giant freshwater prawn |
| S7 | 50-59 | Male | Secondary school | Farm established by the farmer | Giant freshwater prawn |
| S8 | 60-69 | Male | Secondary school | Farm established by the farmer | Pacific white shrimp |
| S9 | 50-59 | Female | Secondary school | Farm inherited | Pacific white shrimp |
| S10 | 40-49 | Female | Bachelor | Farm established by the farmer | Pacific white shrimp, Giant freshwater prawn |
| S11 | 50-59 | Male | Primary school | Farm established by the farmer | Pacific white shrimp |
