# Supplementary material for: Influences on limited antimicrobial use in small-scale freshwater aquaculture farms in central Thailand
Source: Front Vet Sci. 2025 Jul 11;12:1600051. doi: 10.3389/fvets.2025.1600051 (PMC12291687; doi:10.3389/fvets.2025.1600051)
Supplement: SUPPLEMENTARY TABLE S2 — Household characteristics. [file Table_2.docx]

**Supplementary Table S2. Household characteristics**

| Household ID | Household member’s age range | Reported occupations | Aquaculture venture type |
| --- | --- | --- | --- |
| HH03 | 70-79 | Retired. Sells fruits and vegetables from garden: limes, morning glory and lotus stems. Helps feeding the fish in the pond. Makes bamboo baskets for sale and prepares food for the monks. | A fishpond: tilapia, carp, and seven-stripped carp. |
|  | 50-59 | A cook at a school. Helps feeding the fish in the pond. |  |
|  | - | Main caretaker of the fishpond. Works at [University 3] and often comes back on holidays to take care of fishpond. |  |
| HH06 | 40-49 | Delivery driver at [Factory 1] and a fisherman: casts nets in wastewater treatment pond behind the factory and in the river. |  |
| HH07 | 30-39 | Factory worker - fish farmer. | A fishpond: tilapia. It is a family supplementary business. |
|  | - | Factory worker - fish farmer. |  |
| HH14 | 50-59 | Rice farming. Raises chicken for [Poultry farm 1] (contracted, size:3,000 chicken), and raises fish in a pond at home. | A fishpond: tilapia |
| HH19 | 30-39 | A village headman. Has a vegetable garden, a shrimp pond, rice field, and he helps mother at her restaurant | A shrimp pond: around 3-4 rai |
|  | 60-69 | A truck driver. Helps at Family’s restaurant and helps with the pond and the farm. |  |
| HH23 | 50-59 | A Truck driver. The family has some fish in a coop | a cage used for rising fish in ponds or rivers |
| HH25 | - | The family has a chicken farm capacity of a 1000 chicken. They also have a fishpond and they cook and sell food. | A fishpond renovated from a rice field |
| HH27 | 50-59 | A fish and lotus farmer | A fishpond |
|  | 50-59 | A mechanic, fixing agricultural machines. Helps with the pond too |  |
| HH38 | - | A village health volunteer, a farmer (pigs, fish, plants and rice) | A fishpond |
